# Level set segmentation of bovine corpora lutea in *ex situ* ovarian ultrasound images

**DOI:** 10.1186/1477-7827-6-33

**Published:** 2008-08-04

**Authors:** Brennan J Rusnell, Roger A Pierson, Jaswant Singh, Gregg P Adams, Mark G Eramian

**Affiliations:** 1 Department of Computer Science, University of Saskatchewan, Saskatoon, Saskatchewan, Canada; 2 Department of Obstetrics, Gynecology and Reproductive Sciences, University of Saskatchewan, Saskatoon, Saskatchewan, Canada; 3 Department of Veterinary Biomedical Sciences, University of Saskatchewan, Saskatoon, Saskatchewan, Canada

## Abstract

**Background:**

The objective of this study was to investigate the viability of level set image segmentation methods for the detection of corpora lutea (corpus luteum, CL) boundaries in ultrasonographic ovarian images. It was hypothesized that bovine CL boundaries could be located within 1–2 mm by a level set image segmentation methodology.

**Methods:**

Level set methods embed a 2D contour in a 3D surface and evolve that surface over time according to an image-dependent speed function. A speed function suitable for segmentation of CL's in ovarian ultrasound images was developed. An initial contour was manually placed and contour evolution was allowed to proceed until the rate of change of the area was sufficiently small. The method was tested on ovarian ultrasonographic images (*n *= 8) obtained *ex situ*. A expert in ovarian ultrasound interpretation delineated CL boundaries manually to serve as a "ground truth". Accuracy of the level set segmentation algorithm was determined by comparing semi-automatically determined contours with ground truth contours using the mean absolute difference (MAD), root mean squared difference (RMSD), Hausdorff distance (HD), sensitivity, and specificity metrics.

**Results and discussion:**

The mean MAD was 0.87 mm (sigma = 0.36 mm), RMSD was 1.1 mm (sigma = 0.47 mm), and HD was 3.4 mm (sigma = 2.0 mm) indicating that, on average, boundaries were accurate within 1–2 mm, however, deviations in excess of 3 mm from the ground truth were observed indicating under- or over-expansion of the contour. Mean sensitivity and specificity were 0.814 (sigma = 0.171) and 0.990 (sigma = 0.00786), respectively, indicating that CLs were consistently undersegmented but rarely did the contour interior include pixels that were judged by the human expert not to be part of the CL. It was observed that in localities where gradient magnitudes within the CL were strong due to high contrast speckle, contour expansion stopped too early.

**Conclusion:**

The hypothesis that level set segmentation can be accurate to within 1–2 mm on average was supported, although there can be some greater deviation. The method was robust to boundary leakage as evidenced by the high specificity. It was concluded that the technique is promising and that a suitable data set of human ovarian images should be obtained to conduct further studies.

## Background

The ovaries of all mammalian species, including humans and cattle, contain follicles and corpora lutea (CL). The bovine model for studying human ovarian function is well established [1].

### Objective and motivation

**Figure 1:**
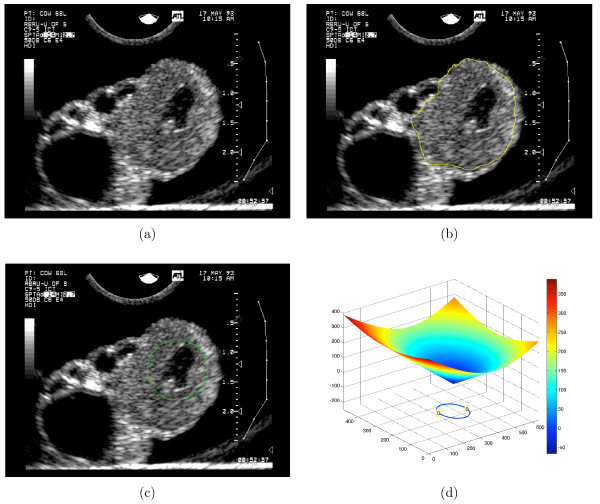
**Corpus Luteum segmentation goal and contour initialization**. (a) Unprocessed ultrasound image containing a corpus luteum; a small irregularly shaped cystic cavity is visualized within the CL. (b) Manual segmentation of the CL by a human expert. (c) A circular initial contour placed within the CL in the image in (a). (d) The surface (*ψ*) of the initial signed distance function in which the contour is embedded.

The objective of this research is to study the viability of level set image segmentation techniques [2] for automatic segmentation of CL (delineation of the boundary) in two-dimensional (2D) ultrasound images. Our study represents the first investigation of semi-automated segmentation of corpora lutea from ultrasound images. The level set method lends itself to image segmentation tasks because it requires minimal user input, accommodates arbitrary changes in region topology and offers a straightforward extension to higher dimensional data [3]. Figure 1(a) shows an image of a CL and Figure 1(b) shows the desired segmentation result as drawn by a human expert in ovarian ultrasound interpretation.

The presence (or absence) of a CL in the ovaries, its size and morphology provide information regarding the current state of the individual's reproductive cycle [4,5]. The most common method of visualizing the CL *in vivo *is ultrasonography. Monitoring the development of ovarian follicles and CL over time is crucial to the understanding of human and bovine reproductive biology, fertility and timing of fertility therapy, the effects of contraception, and the diagnosis of ovarian diseases and dysfunction, such as ovarian cysts, cancers, and polycystic ovarian syndrome.

In practice, CL segmentation is performed manually. Manual delineation is time consuming and subject to variance in human interpretation of images. The domain of image processing offers the potential for detailed analysis of CL size, and appearance, which will facilitate study of the aforementioned processes and diseases. Successful automation of CL segmentation would further the automation of existing analyses, such as correlating the value of image pixels within the CL region with various physiological attributes [6] and automatically determining CL diameter for use in higher-level classifiers [5], as well as facilitate new investigations.

### Literature review

Research on prostate segmentation from 2D ultrasound images was reviewed for insight into CL segmentation due to the similarities of the two problems. The prostate has a similar echotexture appearance to that of corpora lutea in ultrasound images and a similar level of contrast with the rest of the image. Moreover, there is only one region of interest in both problems. However, there are differences which make the CL segmentation problem more challenging. Due to standardized imaging procedures, the prostate's position in an image is approximately known *a priori *and strong assumptions may also be made about the prostate's shape in the image plane [7-10]. This is not possible when imaging corpora lutea since it can be present at different locations within the ovary and is more variable in shape and size [11,12]. CLs are assumed only to be approximately elliptical in the image plane with low to moderate eccentricity. Segmentation of the prostate from ultrasound images has been well studied [7]. A number of prostate segmentation methods used deformable contour models. Deformable contour models were first introduced as tools for image segmentation by Kass et al. [13]. Their active contour models or "snakes" formulated image segmentation as an energy minimization problem. An active contour was initially placed on an image. Solving energy equations caused the curve to move or "evolve" until it minimized the energy function. The energy function was chosen so that the curve tended to follow edges in the image. Snakes were found to poorly handle topological changes in contours such as merging or splitting but the difficulties can be overcome with care, albeit considerable computational overhead.

The success of prostate segmentation using deformable models using snakes and so-called "discrete dynamic contours" were largely dependent on careful initialization of the contour in a position near the desired boundary [14-16].

Badiei et al. developed an algorithm in which small number of marker points were placed on the prostate boundary which was then warped, based on an *a priori *shape model, so that the prostate was elliptical in shape [8]. The marker points were then used to find the ellipse that best fit the warped prostate. The inverse of the warping transformation was applied to the ellipse to obtain the final segmentation. The success of the approach was dependent on the correctness of the assumption of elliptical shape and the careful placement of the initial marker points.

Level set methods has been proven effective both in general [17-20] and for ultrasound image segmentation [21,22]. Recent applications of the level set method to prostate ultrasound image segmentation [3,7,23] show that this technique is accurate and flexible (it can handle contours of varying shape, size, and concavity). A level set method was chosen for the current study because of the prior success of level set methods in prostate segmentation and its ability to easily handle arbitrary changes in contour topology.

Fan et al. [3] used a level set method to perform three-dimensional (3D) surface detection from ultrasound images of the prostate. A fast discrimination method was used to roughly extract the prostate region. The prostate region information was incorporated into the level set method instead of the spatial image gradient. This addressed the issue of "boundary leaking" which occurs when the contour evolves across a very weak edge that is part of the desired boundary. The segmentations produced qualitatively appeared to be good, but a quantitative assessment of segmentation accuracy was lacking.

Herein, a new methodology was created to segment corpora lutea from 2D ultrasound images using this work as a starting point. Our algorithm consists of a multi-step preprocessing stage followed by segmentation using a level set method. It is semi-automated in that it requires an initial user-specified closed contour that is assumed to be completely contained within the CL, and to completely contain the CL's central cavity if one exists. It was hypothesized that this level-set contour evolution method could be used to locate the boundaries of CL with an average error of 1–2 mm. The CLs used in this study ranged from 14.3 mm to 21.5 mm in diameter.

## Methods

### Image data

The work herein is motivated by the need to study human reproduction but images of bovine ovaries were used in this feasibility study as they are well-established as a vehicle for studying human ovaries due to similarities in physiology and morphology. The images used in this study were obtained during a previous study Singh et al. [6]. Left and right ovaries of heifers were surgically removed at defined stages of the estrous cycle and imaged *ex situ *in parallel planes at 0.5 mm increments using a broad-band (5–9 Mhz), convex-array, ultrasound transducer interfaced with an ATL Ultra Mark 9 HDI ultrasound machine (Advanced Technology Laboratories, Brothell, WA). At the time of ovariectomy, the number of days from ovulation was known. From this data set, ovaries with CLs (*n *= 8) were selected for the current study. From the set of parallel images of each ovary, the image which contained the CL at maximum diameter was selected. All images were 640 × 480 pixel 8-bit grayscale, but the pixel size varied across images, ranging from 0.057 mm × 0.057 mm to 0.087 mm × 0.087 mm as determined from the distance gradations on the right-hand side of each image. An experienced gynecologic ultrasonographer manually segmented the images which provided a "ground truth" for validation purposes. The diameters of the CLs in the selected images ranged from 14.2 mm to 21.5 mm. Diameter was estimated by averaging the length and width of the smallest bounding box aligned to the image coordinate axes of the expertly segmented CL region. A sample image is shown in Figure 1(c) and is used as a running example throughout this paper.

### Curve evolution with level sets

In this section, the level set method is presented in a similar fashion to that in [3] which is, in turn, derived from [2]. The level set method evolves an initial 2D contour according to an energy function derived from image pixel data by embedding the 2D contour in a 3D surface.

An initial 2D contour *C*(*t *= 0) is represented by a 3D function *ψ *that evolves over time. The value of *ψ *at a point *p *at time *t *= 0 is defined as the distance *d *from *p *to the nearest point on *C *at time *t*:

(1) *ψ*(*p*, *t *= 0) = ± *d*.

The sign of *d *is negative if the point lies in the interior of *C *and is positive otherwise. The function *ψ*(*p*, *t *= 0) is called a *signed distance function*. Figure 1(c) illustrates a circular initial contour that was used to segment the image on which the contour was superimposed. The corresponding signed distance function, *ψ*(*p*, *t *= 0) is in Figure 1(d); the 2D contour is drawn beneath the surface.

The level set method causes the contour *C*(*t *= 0) to move towards the desired boundary by evolving the surface *ψ *over time. At an aribitrary time *t*, the contour *C*(*t*) is represented by the set of points for which *ψ*(*p*, *t*) = 0, also called the *zero-level set *of *ψ*:

(2) *C*(*t*) = {*p *| *ψ*(*p*, *t*) = 0}.

The evolution of *ψ *is described by the partial differential equation

(3) $$\frac{\partial \psi (p,t)}{\partial t}+F\left|\nabla \psi \right|=0.$$

**Figure 2:**
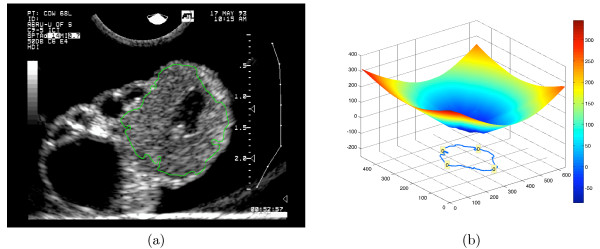
**Result of the level set method**. (a) The contour in Figure 1(c) after evolution with Equation 3 and an appropriate speed function. (b) The corresponding surface.

In this equation, the initial condition is *ψ*(*p*, *t *= 0), ∇*ψ *denotes the gradient of *ψ*, and *F *is the *speed function*. The speed function describes the rate at which the contour will move in the outward normal direction. The contour is encouraged to evolve towards the desired image boundary by designing an appropriate speed function *F*. Figure 2(a) is the contour from Figure 1(c) resulting from evolution using Equation 3 and an appropriate speed function; the corresponding final surface and its zero level set are depicted in 2(b).

In general, the speed function *F *depends on the curvature *K *of the evolving front and is typically separated into a constant term *F*_0 _and the remainder *F*_1_(*K*):

(4) *F*(*K*) = *F*_0 _+ *F*_1_(*K*).

The constant term *F*_0 _provides a constant expansion or contraction force depending on its sign. The curvature-dependent component *F*_1 _controls the smoothness of the deforming shape. A common choice of *F *is given in the following equation:

(5) *F*(*K*) = 1 - *ϵK*.

Equation 5 describes an outward unit normal force that is reduced by a factor proportional to the local curvature of the contour. Intuitively, this causes the non-smooth segments of the contour (which have high curvature) to move slowly while nearby portions of contour "catch up", resulting in smoother curve. The constant *ϵ *regulates the smoothness of the curve and must be greater than zero [3]. Larger values of *ϵ *result in smoother contours.

To cause the evolving contour to stop at the desired image boundary, *F *is typically multiplied with an image dependent quantity *k*_*I*_:

(6) $$k_{I}(x,y)=\frac{1}{1+{\left|\nabla G_{\sigma }*I(x,y)\right|}^{p}},p=1,2.$$

The term ∇*G*_*σ *_denotes the gradient of a two-dimensional Gaussian function whose standard deviation is *σ*, *I *is the image function, and * denotes convolution. The convolution operation is the same as that described for sticks filters (below); only the kernel differs. Since the gradient operator can be applied after the convolution operation without changing the result, the second term of the denominator's sum can be viewed as magnitude of the gradient of an image which has been convolved with a Gaussian filter kernel raised to the power *p*. A Gaussian filter has the effect of computing for each pixel a center-weighted average of the intensities in its local neighborhood which smoothes or blurs the image. A smoothed image is desirable since it reduces the magnitude of small, unimportant local edges so that they have less effect on the speed function.

Pixels with large image gradient (corresponding to pixels that have a high probability of being edges of the corpus luteum) will cause the value of *k*_*I *_(Equation 6) to be close to zero. When *k*_*I *_is multiplied with *F *the speed at which the contour embedded in *ψ *propogates is reduced to nearly zero when it is near the desired boundary. The exponent *p *controls the severity of the penalty that gradient magnitude applies to the speed function.

### Speed function for CL segmentation

The low intensity contrast between CL regions and other regions of the ovary and the noisy nature of ultrasound images required a more sophisticated construction of the speed function, rather than direct application of the process described by Equation 6. The speed function was constructed by filtering the image with a sticks filter [24,25]. The filtered image was then processed with Sobel filters [26] to obtain a gradient magnitude which was then contrast enhanced. The mean image intensity was then subtracted from each pixel and the resulting image was contrast enhanced a second time and inserted into Equation 6 to obtain the weighting term *k*_*I *_for the speed function *F*, above. These steps are discussed in greater detail below.

#### Sticks filter

**Figure 3:**
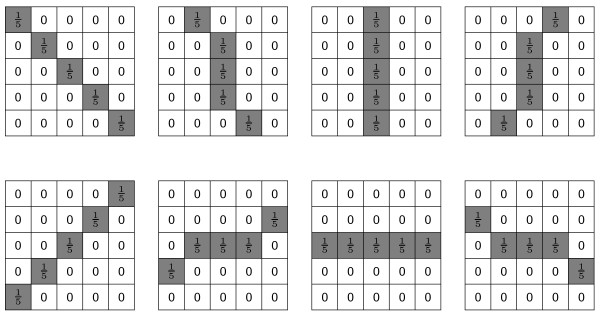
Representation of a sticks filter bank for sticks of length 5.

**Figure 4:**
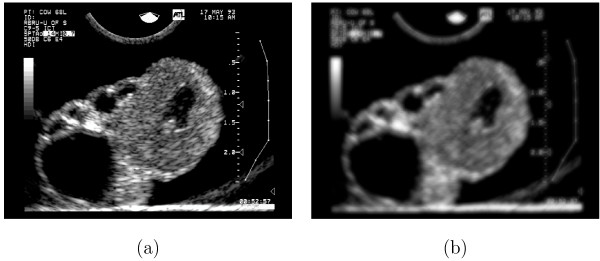
**Effect of the Sticks Filter**. (a) An unprocessed ultrasound image of a corpus luteum. (b) The image in (a) processed with a sticks filter bank of size 17.

Sticks filtering [24,25] is a process for reducing image speckle which may obfuscate boundaries of structures in the image. A sticks filter is a bank of linear filters which evaluate the likelyhood of a linear feature of length *N *in one of 2*N*-2 possible orientations passing through each pixel. Each filter mask is a *N *× *N *matrix where each entry is either 0 or 1/*N*. Entries with value 1/*N *represent lines through the center of the matrix. A bank of filters for *N *= 5 is illustrated graphically in Figure 3. Linear filtering [26] (convolution) works by positioning the filter mask's center over each image pixel and computing the sum of products of each entry in the filter mask with the intensity of the underlying pixel. Each filter mask or "stick" will thus produce the greatest response (sum of products) when positioned over a bright linear feature of a specific orientation. A pixel's output intensity is the response of the filter from the sticks filter bank which responds maximally at that point; the magnitude of the maximal response corresponds to the mean intensity along the line segment orientation to which that filter is most sensitive (the filter response is the sum of the intensities along the stick divided by N). Figure 4(b) shows the effect of sticks filtering on the image in Figure 4(a) using sticks of length 17. The sticks filter suppresses much of the speckle while maintaining the visibility of thin linear features which would be seriously degraded by standard image smoothing techniques.

The first step in our algorithm was to filter the images with a sticks filter bank of size 17. This stick length was chosen because it is small enough that it correlates well with CL boundary segments of the same length but is large enough that it smoothes small-scale features and reduces the magnitude of edges with high curvature which are not of interest. Reducing the magnitude of high-curvature edges is important since the level set method relies heavily on edge information and segmentations are undesirably influenced by small-scale edges such as those caused by speckle.

#### Sobel magnitude filter

Sobel convolution filters are edge detectors which approximate partial first derivatives of the image. The horizontal (*S*_*h*_) and vertical (*S*_*v*_) Sobel filter kernels are

(7) $$\begin{array}{cc} S_{h}=\left[\begin{array}{ccc} 1 & 0 & -1\\ 2 & 0 & -2\\ 1 & 0 & -1 \end{array}\right] & S_{v}=\left[\begin{array}{ccc} 1 & 2 & 1\\ 0 & 0 & 0\\ -1 & -2 & -1 \end{array}\right] \end{array}.$$

These filters compute center-weighted average finite differences over a distance of 3 pixels in a 3 pixel-wide band in the horizontal and vertical directions, respectively.

An edge magnitude image *M *was computed by applying horizontal and vertical Sobel edge detectors to the sticks filtered image and combining the horizontal and vertical responses (respectively *R*_*h *_and *R*_*v*_) in the usual way:

(8) $$M(x,y)=\sqrt{R_{h}{\left(x,y\right)}^{2}+R_{v}{\left(x,y\right)}^{2}}.$$

The Sobel filters may be generalized to larger sizes:

(9) $$\begin{array}{cc} S_{h}=\left[\begin{array}{ccccc} 1 & 0 & \cdots & 0 & -1\\ \vdots & 0 & & 0 & \vdots \\ 2 & \vdots & & \vdots & -2\\ \vdots & 0 & & 0 & \vdots \\ 1 & 0 & \cdots & 0 & -1 \end{array}\right] & S_{v}= \end{array}\left[\begin{array}{ccccc} 1 & \cdots & 2 & \cdots & 1\\ 0 & 0 & \cdots & 0 & 0\\ \vdots & & & & \vdots \\ 0 & 0 & \cdots & 0 & 0\\ -1 & \cdots & -2 & \cdots & -1 \end{array}\right].$$

Larger Sobel filters compute the finite differences over longer distances and bands and are much less sensitive to very local changes. The effect is that larger filters respond to larger scale changes in the image function and small scale changes are not captured.

**Figure 5:**
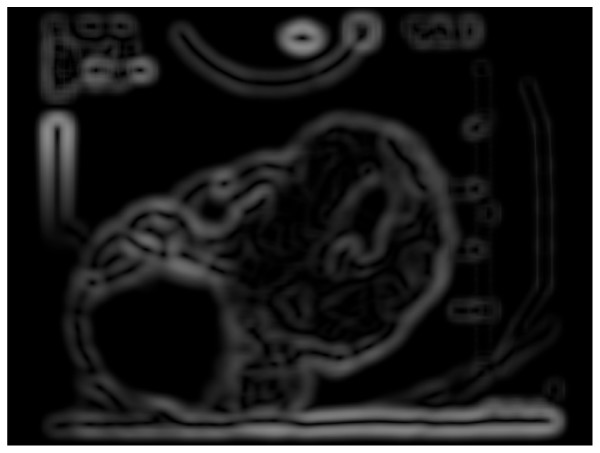
**Effect of edge detection**. This figure shows the result of applying a 17 × 17 Sobel magnitude filter to Figure 4(b). The dominant edges identified at this stage are those more likely to be contours of the corpus luteum.

The use of the Sobel filter at this point in the algorithm was to identify large scale step edges in the image which are characteristic of the CL-stroma boundary. A Sobel filter size of 17 × 17 was chosen empirically by testing the filters on sample images, where preference was given to images that exhibited strong edges along the true boundary of the CL. A size of 17 × 17 enhanced only the major, dominant edges, which are more likely to be true boundaries of the CL. Figure 5 shows the results of processing Figure 4(b) with a 17 × 17 Sobel magnitude filter.

#### Intensity normalization

In ultrasonographic images, even the strongest edges may have fairly weak magnitude. In order to improve the contrast and further emphasize major edges, the pixel intensities of the edge magnitude image *M *were normalized to obtain the image $\bar{M}$.

Normalization is a linear remapping of pixel intensities. If the pixel intensities in an image are within the interval [*I*_*min*, _*I*_*max*_], then a normalizing linear remapping causes intensity *I*_*min *_to be mapped to intensity 0, and *I*_*max *_to be mapped to the maximum possible intensity while intensities between *I*_*min *_and *I*_*max *_are linearly interpolated. After normalization, images were converted from 8-bit grayscale (integer pixel intensities ranging from 0 to 255) to real-valued images with intensities ranging from 0.0 (black) to 1.0 (white) by dividing each pixel's intensity by 255.

#### Weak edge suppression

**Figure 6:**
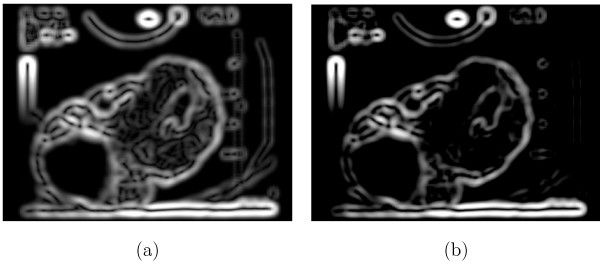
**Removal of weak edges within the CL**. (a) The normalized image from Figure 5. (b) The image from (a) after subtraction of global average, truncation, and re-normalization; the weak edges within the CL have been largely suppressed.

The normalized edge magnitude image obtained in the previous step exhibits weak edges within the interior of the CL region (Figure 6(a)) caused by the fine texture present there. These edges interfere with the level set segmentation process. Weak edges were suppressed by subtracting from each pixel in the normalized edge magnitude image the mean intensity of the normalized edge magnitude image and clipping all resulting negative intensities to zero. Formally, let *μ *be the mean intensity of the normalized edge magnitude image $\bar{M}$. The mean-subtracted edge magnitude image *N *is defined as

(10) $$N(x,y)=\left\{\begin{array}{lll} \bar{M}(x,y)-\mu & : & \bar{M}(x,y)-\mu >0;\\ 0 & : & otherwise. \end{array}\right\}$$

The resulting image *N *was re-normalized using the procedure described above to obtain the normalized mean-subtracted edge magnitude image $\bar{N}$. Figure 6(b) illustrates the effect of mean subtraction from the normalized edge magnitude image in Figure 6(a); the weaker edges present in the CL region have been largely suppressed. The central fluid cavity in the pictured CL causes the high-magnitude edge that is retained in the renormalized image. The image $\bar{N}$ was used to compute the weighting term *k*_*I *_for the speed function *F *in the level set method.

### Level set segmentation of corpora lutea

#### Placement of the initial contour

Placement of the initial 2D contour was done manually. All initial contours were circular. If a fluid cavity was present, the initial contour was chosen to enclose the fluid cavity. Kastelic et al. showed that 79% of bovine ovaries exhibit central cavities [27]. If a fluid cavity was not present, the contour was placed near the center of the CL. The remainder of the algorithm is automatic.

The intial 2D contour was embedded as the zero level set of a 3D signed distance function *ψ*(*p*, *t *= 0). This function was evolved using Baris Sumengen's Matlab level set toolbox [28] according to Equation 3.

#### Curve evolution parameters

The weighting term *k*_*I *_for the speed function F was computed from the preprocessed image $\bar{N}$ in the previous section using a slightly altered version of Equation 6:

(11) $$k_{I}(x,y)=\frac{1}{1+\alpha \times {\left[\bar{N}(x,y)\right]}^{p}}.$$

In order to achieve finer control over the rate at which *k*_*I *_approaches zero as $\bar{N}$ increases, a coefficient *α *was added to the second term of the denominator.

**Figure 7:**
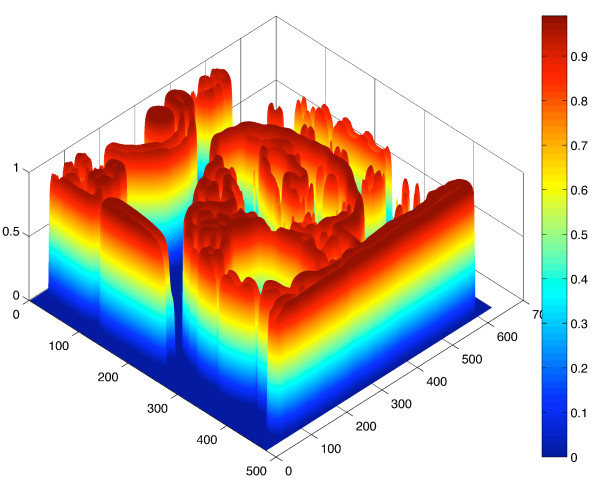
**Visualization of the speed function**. A surface plot of 1 - *k*_*I *_(inverted for ease of interpretation) computed by using the image in Figure 6(b) for $\bar{N}$ in Equation 11. Areas close to the desired contour of the corpus luteum boundary are red in colour, indicating that the evolution of *ψ *will slow and eventually stop near the true contour of the corpus luteum.

Values of *α *= 100 and *p *= 1 were chosen empirically. A surface plot of 1-*k*_*I *_(inverted for ease of interpretation) computed using the image in Figure 6(b) as $\bar{N}$ is shown Figure 7. Strong edges are red in colour, indicating that the evolution of *ψ *will slow and eventually stop near the true contour of the CL. The parameter *ϵ *in Equation 5 was experimentally chosen to be 0.375. Use of a non-zero curvature-based force was used to discourage the "leaking" of the evolving contour through sections of the true CL boundary where the magnitude of the edge was weak.

The Matlab toolbox [28] which was used to evolve the boundary also required some additional parameters. The first-order accurate finite difference option was chosen for approximation of derivatives. The surface being evolved must be discretized on a grid; a conservative grid size of 1 × 1 was chosen. The time interval between steps of the evolution, which may range from 0.5–0.9, was chosen to be a conservative 0.5. A more aggressive selection of a larger timestep would improve the speed of the curve evolution algorithm, but could also cause numeric instability leading to poor or nonsensical results.

The surface in which the 2D contour is embedded will almost certainly lose the property of being a signed distance function as it evolves since portions of surface will move at different velocities. It is necessary to periodically re-initialize the surface to a signed distance function, for example, after every *m *time steps. This is achieved by extracting the zero-level set *C*(*t*) (defined in Equation 2) and constructing from it a new signed distance function *ψ*_*new*_. The evolution process then continues using *ψ*_*new *_for another *m *time steps. This is repeated until a stopping condition is condition is met (see below). A value of *m *= 1 was selected which caused the surface to be reinitialized as a signed distance function after every time step. This ensured that the surface was always a signed distance function and facilitated the test of the stopping condition.

#### Stopping condition

The stopping condition determined when to cease evolution of *ψ*. Since the zero level set contour will presumably stop when it has settled onto the desired boundary, a test is needed to determine when motion of *C*(*t*) becomes sufficiently small. The criterion used was motivated by the work of [29]: "If the iteration count [total number of time steps] *n *exceeds a particular threshold *N*_0 _and the rate of change of contour length $\left|\frac{dL_{c}(t)}{dt}\right|$ <*γ *over a fixed number of iterations Δ*n*, STOP the iteration process".

Herein, *L*_*c*_(*t*), the length of the contour *C*(*t*), was replaced with the area of the interior of *C*(*t*), denoted *A*_*c*_. The quantity $\left|\frac{dA_{c}(t)}{dt}\right|$ was computed at each time step and a record of these values for the previous Δ*m *time steps was maintained. The evolution was halted when *n *> *N*_0 _and $\left|\frac{dA_{c}(t)}{dt}\right|$ <*γ *was true for all of the previous Δ*m *time steps. Values *N*_0 _= 250 and *γ *= 250, and Δ*m *= 15 were used to evaluate the stopping condition.

**Figure 8:**
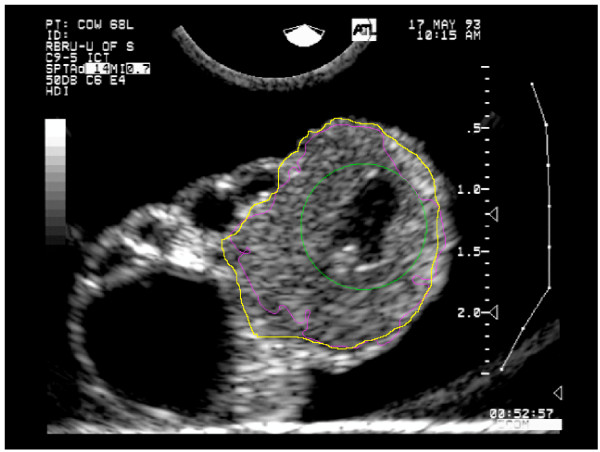
**Result of segmentation**. The contour detected by the proposed method using the image and initial contour from Figure 1(c) as input to the level set method. The green contour is the initial contour, the purple contour is the final (automatic) contour, and the yellow contour is the contour determined by an experienced human interpreter.

Figure 8 shows the contour detected by the proposed method using the lower CL image from Figure 1(a) as input to the level set method. The green contour is the initial contour, the purple contour is the final (automatic) contour, and the yellow contour is the contour determined by an experienced human interpreter. Results of the method on the entire data set are discussed in the Results section, below.

#### Smoothing with Fourier descriptors

During development of the implementation of the above algorithm, it was observed that the region boundaries determined by the algorithm quite often meandered back and forth across the expertly traced boundary. It was hypothesized that if the boundaries were smoothed, they might conform more closely to the ground truth boundary. Boundaries were smoothed by truncating the Fourier descriptors of the boundary [26]. This section reviews the basics of smoothing using Fourier descriptors.

Let *P *= (*p*_1_,...,*p*_*B*_) be a sequence of *B *points representing the pixel locations of a boundary in (anti-)clockwise order. For the *i*-th point, *p*_*i*_, let *x*(*p*_*i*_) and *y*(*p*_*i*_) respectively denote the *x *and *y *coordinates of *p*_*i*_. The boundary can then be represented as a sequence of complex numbers *S *= (*s*_1_,*s*_2_,...,*s*_*B*_) where *s*_*i *_= *x*(*p*_*i*_) + *jy*(*p*_*i*_) and *j *= $\sqrt{-1}$. The *Fourier descriptors*, *a*(*u*), of the boundary are the coefficients of the Fourier transform of *S*:

(12) $$a(u)=\frac{1}{B}\sum_{i=0}^{B}s_{i}e^{-j2\pi ui/B}.$$

The original boundary may be recovered via the inverse Fourier transform. However, *S*, (and, in turn, *P*) may be approximated by using only the first *Z *≤ *B *Fourier descriptors in the computation of the inverse transform:

(13) $${\hat{s}}_{i}=\sum_{u=0}^{Z-1}a(u)e^{j2\pi ui/B}.$$

**Figure 9:**
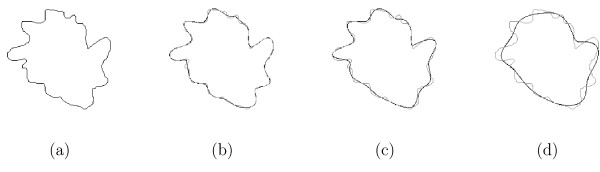
**Effect of smoothing a contour by truncating Fourier descriptors**. (a) An unsmoothed meandering contour. (b) The contour in (a) smoothed by retaining only the first 5% of the Fourier descriptors; the smoothed contour is drawn in black and the original contour is superimposed in grey. (c) Retaining only the first 3% of descriptors. (d) Retaining only the first 2% of descriptors, the smoothed contour now follows the "average" path rather than meandering.

This acts as a "lowpass filter" on the contour, and reduces the magnitude of high frequency variations which results in a smoother contour. The fewer descriptors that are retained, the greater the smoothing effect, as illustrated in Figure 9. The effects of smoothing on contour accuracy are discussed in the Results section.

### Validation

The manual segmentations obtained for this study were used as the basis for comparison when validating the semi-automatic segmentations. The metrics [30,31] mean absolute distance (MAD), root mean squared distance (RMSD), Hausdorff distance (HD), sensitivity, and specificity were used to evaluate segmentation accuracy. These metrics are formally defined below.

The minimum distance *d*_*min *_between a point *p*, and a contour *C*, is defined as

(14) $$d_{min}(p,C)=\underset{q\in C}{\min}||p-q||,$$

which is the smallest Euclidean distance between *p *and any point on the contour *C*.

Let *C*_*M *_and *C*_*A *_denote the sets of points on the manually segmented and semi-automatically segmented contours, respectively. The mean absolute distance between *C*_*M *_and *C*_*A *_is

(15) $$MAD=\frac{1}{\left|C_{M}\right|}\sum_{p\in C_{M}}d_{min}(p,C_{A})$$

where |*C*_*M*_| denotes the number of points in the set *CM*. Smaller MAD indicates a better segmentation. The root mean squared distance between *C*_*M *_and *C*_*A *_is

(16) $$RMSD=\sqrt{\frac{1}{\left|C_{M}\right|}\sum_{p\in C_{M}}{\left[d_{min}(p,C_{A})\right]}^{2}}.$$

This metric is similar to MAD, except that pixels in *C*_*A *_which are further away from *C*_*M *_contribute a greater penalty to the metric.

Hausdorff distance is the greatest minimum distance between two contours. It characterizes the maximum deviation of one contour from another. Formally,

(17) $$HD=\max\left[\underset{p\in C_{M}}{\max}d_{min}(p,C_{A}),\underset{q\in C_{A}}{\max}d_{min}(q,C_{M})\right].$$

A smaller HD is more desirable.

Let *TP *denote the set of *true positive *pixels, that is, those pixels that were correctly identified as belonging to a CL region. Let *TN *be the set of *true negative *pixels correctly identified as not belonging to a CL region. Similarly define *FP *and *FN *to be the set of *false positive *and *false negative *pixels. The sensitivity of a segmentation is thus defined as

(18) $$sensitivity=\frac{\left|TP\right|}{\left|TP\right|+\left|FN\right|}$$

and represents the percentage of pixels that truly belong to the CL region which were correctly identified as such. The specificity of a segmentation is the ratio

(19) $$specificity=\frac{\left|TN\right|}{\left|TN\right|+\left|FP\right|}.$$

Specificity represents the percentage of pixels which are not part of the CL region which were correctly identified as such. A perfect segmentation with respect to the ground truth will have both a sensitivity and a specificity of 1.0.

## Results

The eight images in our testing set were segmented with the algorithm described above. The manual segmentations performed by a human expert were compared with the automated segmentations by computing the previously described metrics for each image.

Table 1 summarizes the validation metrics for each image. Overall, the mean MAD was 0.87 mm (*σ *= 0. 36 mm), the mean RMSD was 1.1 mm (*σ *= 0.47 mm), mean Hausdorff distance was 3.4 mm (*σ *= 2.0 mm), mean sensitivity was 0.814 (*σ *= 0.171), and mean specificity was 0.990 (*σ *= 0.00786).

**Table 1:** Quality of the CL segmentations analyzed by computing the absolute distance (MAD), root mean square distance (RMSD), and Hausdorff distance (HD) between the automatically and manually determined contours.

Image ID	MAD (mm)	RMSD (mm)	HD (mm)	Specificity	Sensitivity
1	0.61	0.79	2.3	0.986	0.938
2	1.5	1.9	5.7	0.996	0.654
3	0.60	0.74	1.9	0.989	0.916
4	0.77	1.0	3.0	0.979	0.914
5	0.68	0.87	2.1	0.980	0.884
6	1.4	1.8	7.3	1.00	0.457
7	0.77	1.0	2.6	0.998	0.834
8	0.65	0.82	2.2	0.990	0.915

Mean ± *σ*	0.87 ± 0.36	1.1 ± 0.47	3.4 ± 2.0	0.990 ± 0.00786	0.814 ± 0.171

**Figure 10:**
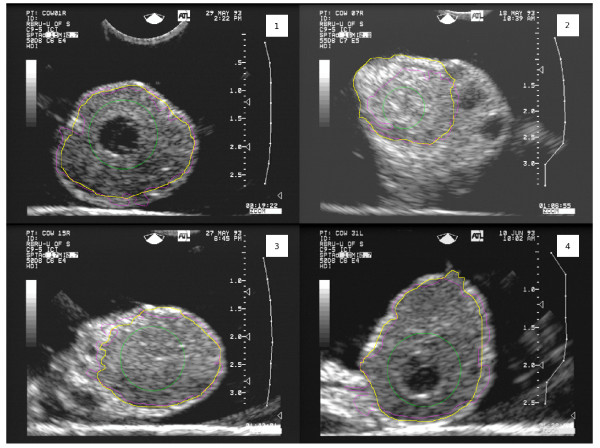
**Segmentation results for test images 1–4**. The green contours are the initial contours, the purple contours are the final (automatic) contours, and the yellow contours are the contours determined by a human expert. The image numbering corresponds to "Image ID" in Table 1.

**Figure 11:**
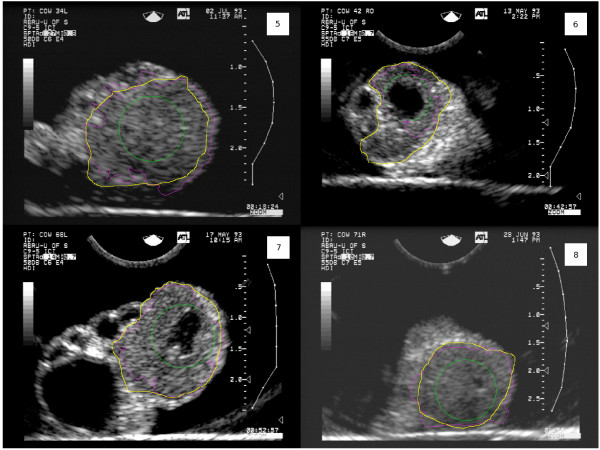
**Segmentation results for test images 5–8**. The green contours are the initial contours, the purple contours are the final (automatic) contours, and the yellow contours are the contours determined by a human expert. The image numbering corresponds to "Image ID" in Table 1.

Figures 10 and 11 show the results of segmentation for all test images. The green contours are the initial contours, the purple contours are the final automatically determined contours, and the yellow contours are the contours manually determined by a human expert.

The mean runtime for the completion of the segmentation of one image is 5 min 54s (*σ *= 1 min 41s). All test images were segmented using a 2.80 GHz Pentium 4 Processor with 2 GB of RAM running Mandriva Linux (2.6.17–14 mdv). All segmentations were executed using MATLAB (The Mathworks Inc., Natick, MA, USA) version 7.4.0.336 (R2007a).

Smoothing with Fourier descriptors was performed on the automatically segmented contours. Table 2 shows the mean MAD, mean RMSD, mean HD, specificity, and sensitivity over all eight test images while varying the percentage of descriptors used to construct the smoothed contour. Table 2 demonstrates that as the percentage of descriptors was decreased, all validation metrics improved. However, the improvements were negligible; the greatest improvement was in the RMSD metric using 1% of the Fourier descriptors, where the mean RMSD decreased by 0.2 mm.

**Table 2:** Effect of smoothing by truncation of Fourier descriptors on mean quality metrics as a function of percentage of Fourier descriptors retained.

Percentage Used (%)	MAD (mm)	RMSD (mm)	HD (mm)	Specificity	Sensitivity
100 (no smoothing)	0.87 ± 0.36	1.1 ± 0.47	3.4 ± 2.0	0.990 ± 0.00786	0.814 ± 0.171
10	0.87 ± 0.36	1.1 ± 0.47	3.4 ± 2.0	0.990 ± 0.00784	0.814 ± 0.171
7.5	0.86 ± 0.35	1.1 ± 0.46	3.4 ± 2.0	0.990 ± 0.00782	0.815 ± 0.171
5	0.86 ± 0.35	1.1 ± 0.46	3.4 ± 2.0	0.990 ± 0.00778	0.815 ± 0.172
2.5	0.83 ± 0.35	1.1 ± 0.47	3.3 ± 2.1	0.991 ± 0.00751	0.817 ± 0.175
1	0.77 ± 0.43	0.95 ± 0.54	3.1 ± 2.1	0.992 ± 0.00654	0.821 ± 0.184

## Discussion

Table 1 and Figures 10 and 11 demonstrate quite clearly that the proposed algorithm typically under-segments CL; specificity is generally excellent, while sensitivity varies from very good (0.938) to quite poor (0.457). The high specificity occurred because the contours were initialized inside the CL and grew outward. The sensitivity was generally lower because of the noisy nature of the images. The gradient information captured in *k*_*I *_(Equation 11) did not perfectly match the true contour of the CL which caused the curve evolution to stop too soon in many instances. There are no other CL segmentation algorithms with which this work could be compared. Given that the mean Hausdorff distance is only 3.4 mm (*σ *= 2.0 *mm*) and the images from Figures 10 and 11, it is clear that the regions are being under-segmented, but the degree of under-segmentation is fairly uniform about the true contour. Smoothing with Fourier descriptors resulted in a minor improvement, but it is concluded that the additional computation is not worth the degree of improvement. The advantages of the algorithm are that it can handle arbitrary contours and requires no user intervention beyond placement of the initial contour. Moreover, no serious boundary leaking occurred resulting in very high specificity in the segmentations. Though similar to the work of Gooding et al. [32], our work differs in that a substantial amount of preprocessing is needed to enhance the gradient formed by CL boundaries. Gooding et al. use a similar gradient-based level set contour evolution method to segment ovarian follicles from 3D images. Follicles are easier to distinguish from their environment with gradient-based level set methods without aggressive preprocessing because the gradient at follicle boundaries is usually quite strong.

There are several aspects of this algorithm that could be improved with future work. A combination of texture and intensity properties might be able to locate a small area of the CL with high confidence in which the initial contour could be automatically placed. Recent work on distinguishing CL echotexture from that of ovarian stroma has shown promise [33] and this work will be continued by examining wavelet-based texture features. A challenge in automatic contour placement will be to distinguish CL texture not only from that of stroma, but from that of a corpus albicans which are non-functional and are comprised of more dense tissue.

A more rigorous exploration of the algorithm's parameter space (which is of high dimension) could yield a set of parameters that produces superior results. The speed of the contour evolution is clearly an issue. Speedup by a factor of 2–3 is likely possible by re-implementing the methods herein using a programming language such as C or C++. Real-time speeds are not likely achievable using level-set-based contour evolution, thus, more computationally efficient segmentation algorithms will be investigated to see whether they may be suitable for CL segmentation.

The algorithm must be tested on CL's imaged *in vivo*. It is expected that the algorithm will give similar performance on such images. Since the algorithm was successful in locating boundaries between lutetal tissue and stroma we expect it to perform similarly at boundaries where the protruding CL is closely interfaced with organs and tissues surrounding the ovary. Moreover, our preprocessing causes significant blurring in order to "plug" holes in the CL boundary through which leaks into the background may occur which affects the potential accuracy of boundary location. Images acquired *in vivo *may permit less aggressive preprocessing since the area of the image surrounding the ovary will be textured and not have a near-zero gradient through which the contour can leak. In turn, less blurring would cause less damage to the shape of the boundary, permitting a higher accuracy in boundary location.

The evidence that CL morphology is related to function is conflicting, particularly between different laboratories and species. A study by Tom et al. supported the hypothesis that quantitative changes in luteal echotexture in bovine corpora lutea are indicative of changes in its physiologic status and capacity to elaborate progesterone [34]. In mares, luteal area was positively correlated with circulating progesterone levels; however, the presence of a cystic cavity within the CL did not affect the luteal gland's ability to produce progesterone [27,35]. In humans, mean luteal area of human CL's was shown to be positively correlated with serum progesterone concentrations (*r *= +0.88) and serum estradiol concentrations (*r *= +0.62) [36]. The human study is particularly encouraging with regard to the prospect of non-invasive automated interpretation of physiologic information from images which could obviate blood tests in future generations. In this context, the current study and others of its kind will be crucial to automated, standardized analysis of CL images. The work presented in the present study also is useful for automated recognition of CL in the studies of CL morphology and has the potential to be used in different imaging modalities such as histology, ultrasonography and magnetic resonance imaging [37,38]. Indeed, any study which would benefit from automatic measurements of CL diameter, such as that of Maldonado-Castillo et al. [5], would be an application for automated CL segmentation algorithms.

## Conclusion

This work is a first step towards a fully automated CL segmentation algorithm which is quite successful when the initial contour is manually placed. The hypothesis that our algorithm can locate CL boundaries within 1–2 mm on average is accepted. It is concluded that level set contour evolution methods are a viable option for offline (non-realtime) CL segmentation and warrant further study.

The CL segmentation algorithm herein is a prototype for the currently missing piece of a comprehensive segmentation algorithm for all ovarian structures (follicles, CL, stroma). The work has both research and clinical importance as noninvasive gynecologic imaging becomes more firmly integrated into standard clinical practice. Standardized descriptions and image interpretations are critical in developing standardized diagnostic algorithms and clinical practice guidelines.

## List of abbreviations

CL: Corpus luteum; HD: Hausdorff distance; MAD: Mean absolute distance; RMSD: Root mean squared distance.

## Competing interests

The authors declare that they have no competing interests.

## Authors' contributions

BJR carried out the literature review and experiments, and drafted the initial manuscript. MGE directly supervised the research, is primarily responsible for the design of the study, and is largely responsible for the final draft of the manuscript. RAP participated in writing the introduction, discussion, and conclusion sections of the manuscript and provided "ground truth" for the CL segmentations to be evaluated. GPA and JS provided the image data for the study, advised on the design of the study, and participated in proofreading the manuscript. All authors have read and approved the final manuscript.

## References

[B1] Adams GP, Pierson RA (1995). Bovine model for study of ovarian follicular dynamics in humans. Theriogenology.

[B2] Sethian JA (1996). Level Set Methods and Fast Marching Methods – Evolving Interfaces in Geometry, Fluid Mechanics, Computer Vision and Materials Science.

[B3] Fan S, Voon LK, Ng WS (2002). 3D Prostate Surface Detection from Ultrasound Images Based on Level Set Method. Med Image Comput Comput Assist Interv Int Conf Med Image Comput Comput Assist Interv.

[B4] Baerwald AR, Adams GP, Pierson RA (2003). A new model for ovarian follicular development during the human menstrual cycle. Fertil Steril.

[B5] Maldonado-Castillo I, Eramian M, Pierson R, Singh J, Adams G (2007). Classification of Reproductive Cycle Phase using Ultrasound-detected Features. Proceedings of the 4th Canadian Conference on Computer and Robot Vision, IEEE.

[B6] Singh J, Pierson RA, Adams GP (1997). Ultrasound image attributes of the bovine corpus luteum: structural and functional correlates. J Reprod Fertil.

[B7] Fan S, Voon LK, Ng WS, Wu RY (2003). Prostate Boundary Detection From Ultrasonographic Images. J Ultrasound Med.

[B8] Badiei S, Salcudean SE, Varah J, Morris WJ (2006). Prostate Segmentation in 2D Ultrasound Images Using Image Warping and Ellipse Fitting. Med Image Comput Comput Assist Interv Int Conf Med Image Comput Comput Assist Interv.

[B9] Pathak SD, Chalana V, Haynor DR, Kim Y (2000). Edge-Guided Boundary Delineation in Prostate Ultrasound Images. IEEE Trans Med Imaging.

[B10] Gong L, Pathak SD, Haynor DR, Cho PS, Kim Y (2004). Parametric shape modeling using deformable superellipses for prostate segmentation. IEEE Trans Med Imaging.

[B11] Pierson R, Ginther O (1987). Reliability of Diagnostic Ultrasonography for Identification and Measurement of Follicles and Detecting the Corpus Luteum in Heifers. Theriogenology.

[B12] Pierson R, Ginther O (1988). Ultrasonic Imaging of the Ovaries and Uterus in Cattle. Theriogenology.

[B13] Kass M, Witkin A, Terzopoulos D (1988). Snakes: Active Contour Models. International Journal of Computer Vision.

[B14] Pathak S, Aarnink RG, de La Rosette JJ, Chalana V, Wijkstra H, Haynor DR, Debruyne FM, Kim Y (1998). Quantitative three-dimensional transrectal ultrasound (TRUS) for prostate imaging. Proc Soc Photo Opt Instrum Eng, Bellingham, WA.

[B15] Ladak H, Mao F, Wang Y, Downey D, Steinman D, Fenster A (2000). Prostate segmentation from 2D ultrasound images. World Congress on Medical Physics and Biomedical Engineering.

[B16] Wang Y, Cardinal HN, Downey DB, Fenster A (2003). Semiautomatic three-dimensional segmentation of the prostate using two-dimensional ultrasound images. Med Phys.

[B17] Paragios N, Deriche R (2002). Geodesic Active Regions and Level Set Methods for Supervised Texture Segmentation. International Journal of Computer Vision.

[B18] Malladi R, Sethian JA, Vemuri BC (1995). Shape Modeling with Front Propagation: A Level Set Approach. IEEE Trans Pattern Anal Mach Intell.

[B19] Ranchin F, Dibos F (2005). Variational level set methods: from continuous to discrete setting, applications in video segmentation and tracking. IEEE International Conference on Image Processing.

[B20] Santis AD, Iacoviello D (2005). Discrete level set approach to image segmentation.

[B21] Lin P, Zheng C, Yang Y, Gu J (2004). Medical Image Segmentation by Level Set Method Incorporating Region and Boundary Statistical Information. Progress in Pattern Recognition, Image Analysis and Applications.

[B22] McInerney T, Terzopoulos D (1996). Deformable models in medical image analysis: a survey. Med Image Anal.

[B23] Gong L, Ng L, Pathak SD, Tutar I, Cho PS, Haynor DR, Kim Y, Fitzpatrick MJ, Reinhardt JM (2005). Prostate ultrasound image segmentation using level set-based region flow with shape guidance. Proc Soc Photo Opt Instrum Eng.

[B24] Czerwinski RN, Jones DL, O'Brien WD (1998). Line and Boundary Detection in Speckle Images. IEEE Trans Image Process.

[B25] Czerwinski RN, Jones DL, O'Brien WD (1999). Detection of Lines and Boundaries in Speckle Images – Application to Medical Ultrasound. IEEE Trans Med Imaging.

[B26] Gonzalez RC, Woods RE (2008). Digital Image Processing.

[B27] Kastelic JP, Pierson RA, Ginther OJ (1990). Ultrasonic Morphology of Corpora Lutea and Central Luteal Cavities During the Estrous Cycle and Early Pregnancy in Heifers. Theriogenology.

[B28] Sumengen B (2005). A Matlab toolbox implementing Level Set Methods. http://barissumengen.com/level_set_methods/index.html.

[B29] Chaudhury KN, Ramakrishnan KR (2007). Stability and convergence of the level set method in computer vision. Pattern Recognition Letters.

[B30] Chalana V, Kim Y (1997). A methodology for evaluation of boundary detection algorithms on medical images. IEEE Trans Med Imaging.

[B31] Bowyer KW, Fitzpatrick JM, Sonka M (2000). Validation of Medial Image Analysis Techniques. Handbook of Medical Imaging: Medical Image Processing and Analysis.

[B32] Gooding MJ, Kennedy S, Noble JA (2008). Volume segmentation and reconstruction from freehand 3D ultrasound data with application to ovarian follicle measurement. Ultrasound Med Biol.

[B33] Eramian MG, Adams GP, Pierson RA (2007). Enhancing Ultrasound Texture Differences for Developing an *in vivo *"Virutal Histology" Approach to Bovine Ovarian Imaging. Reprod Fert Develop.

[B34] Tom JW, Pierson RA, Adams GP (1998). Quantitative echotexture analysis of bovine corpora lutea. Theriogenology.

[B35] Townson DH, Pierson RA, Ginther OJ (1989). Characterization of Plasma Progesterone Concentrations for Two Distinct Luteal Morphologies in Mares. Theriogenology.

[B36] Baerwald AR, Adams GP, Pierson RA (2005). Form and function of the corpus luteum during the human menstrual cycle. Ultrasound Obst Gyn.

[B37] Sarty GE, Adams GP, Pierson RA (2000). Three-dimensional magnetic resonance imaging for the study of ovarian function in a bovine *in vitro *model. J Reprod Fertil.

[B38] Hilton JL, Baerwald AR, Sarty GE, Adams GP, Pierson RA (2003). Magnetic Resonance Image Attributes of the Bovine Corpus Luteum During Development and Regression. Anat Rec Part A.

